# Phototrophic sulfide oxidation: environmental insights and a method for kinetic analysis

**DOI:** 10.3389/fmicb.2013.00382

**Published:** 2013-12-19

**Authors:** Thomas E. Hanson, George W. Luther, Alyssa J. Findlay, Daniel J. MacDonald, Daniel Hess

**Affiliations:** ^1^School of Marine Science and Policy, University of DelawareLewes, DE, USA; ^2^Department of Biological Sciences, University of DelawareNewark, DE, USA; ^3^Delaware Biotechnology Institute, University of DelawareNewark, DE, USA; ^4^Department of Chemistry and Biochemistry, University of DelawareNewark, DE, USA

**Keywords:** phototrophic bacteria, sulfide oxidation, Chesapeake Bay, voltammetry, euxinia

## Abstract

Previously, we presented data that indicated microbial sulfide oxidation would out-compete strictly chemical, abiotic sulfide oxidation reactions under nearly all conditions relevant to extant ecosystems (Luther et al., 2011). In particular, we showed how anaerobic microbial sulfide oxidation rates were several orders of magnitude higher than even metal catalyzed aerobic sulfide oxidation processes. The fact that biotic anaerobic sulfide oxidation is kinetically superior to abiotic reactions implies that nearly all anaerobic and sulfidic environments should host microbial populations that oxidize sulfide at appreciable rates. This was likely an important biogeochemical process during long stretches of euxinia in the oceans suggested by the geologic record. In particular, phototrophic sulfide oxidation allows the utilization of carbon dioxide as the electron acceptor suggesting that this process should be particularly widespread rather than relying on the presence of other chemical oxidants. Using the Chesapeake Bay as an example, we argue that phototrophic sulfide oxidation may be more important in many environments than is currently appreciated. Finally, we present methodological considerations to assist other groups that wish to study this process.

## Introduction

**Figure 1:**
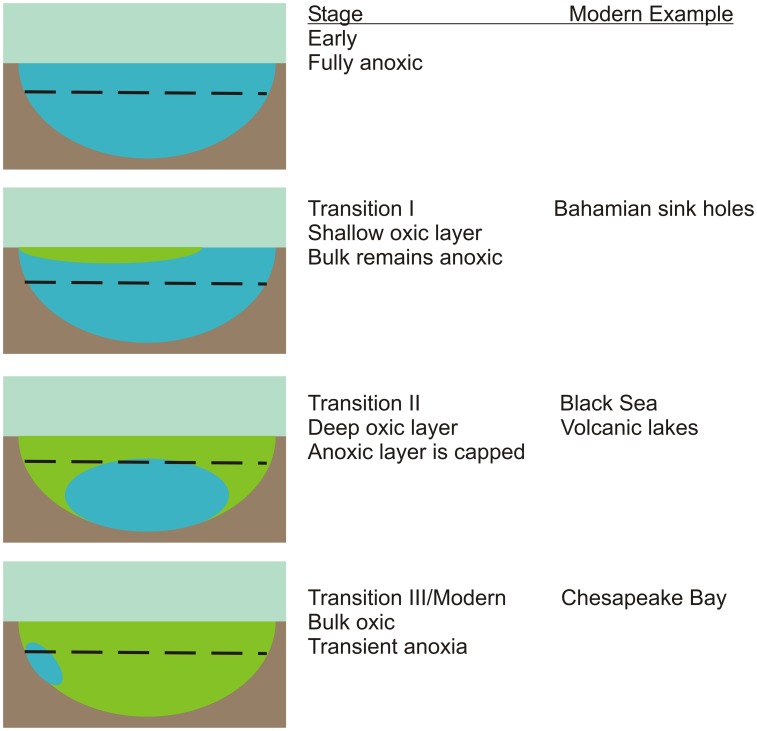
**A simplified conceptual model for the progressive transition from an early anoxic ocean (blue) to modern oxygenated oceans (green)**. The dashed line indicates the depth of the photic zone. The chemistry and microbiology of the transient anoxia that develops in the Chesapeake Bay on an annual basis will be used in this project as a proxy for the last transitional stage. The group has access to samples and data from the Black Sea chemocline and Bahamian sink holes that are thought to represent additional transition stages.

The accumulation of molecular oxygen in Earth's biosphere was not instantaneous with the onset of oxygenic photosynthesis. Rather, it is thought to have occurred over billions of years and not necessarily as a smooth gradual progression of O_2_ concentrations, but as a series of at least two steps (Scott et al., 2008; Frei et al., 2009; Farquhar et al., 2011). Substantial reservoirs of reduced chemical species (i.e., Fe^2+^ and HS^−^) are thought to have existed in early oceans and these would have to be oxidized prior to the full oxygenation of the oceans. Furthermore, as O_2_ production is light dependent, ocean oxygenation and oxidation was most likely a top down process, i.e., shallow layers with highest oxygen production rates would have become oxygenated more rapidly over time than deep water layers (Figure 1). The Archaean ocean was **ferruginous** with low sulfur concentrations (Canfield et al., 2000). The initial rise of oxygen in the atmosphere increased continental weathering thus increasing the flux of oxidized sulfur species to the oceans (Scott et al., 2008). Evidence suggests that resulting sulfate reduction led to a sulfidic ocean being established ~1.8 Gyr ago (Poulton et al., 2004) that persisted until ~0.6–0.5 Gyr ago (Wille et al., 2008; Gill et al., 2011). However, recent data indicate that ferruginous conditions also existed for at least the first half of this time period suggesting a dynamic and variable ocean during this transition.

KEY CONCEPT 1 | Ferruginous Environments characterized by anoxia and the presence of soluble ferrous iron that produces oxides when exposed to oxic conditions.

From this reasoning, it follows that microbial sulfide oxidation in marine systems must have become important after the first flush of sulfate to the oceans and microbial sulfide oxidizers may have been major components of the pelagic marine microbial community for upwards of 1.2 billion years. Furthermore, as oxygen increased in the atmosphere and the oceans, microbial sulfide oxidizers would have experienced drastic changes to their environments that would have required adaptation or extinction. In this review, we speculate on the role of phototrophic sulfide oxidizing bacteria in modern analogs of ancient oceans using the Chesapeake Bay as an illustrative example. We also provide technical details on one method for measuring phototrophic dependent sulfide oxidation. While this is an unusual structure for a review article, we hope to both stimulate interest in the first part of the review and then equip the reader with a tool to help transform interest into practical experiments that will, hopefully, avoid pitfalls in performing and interpreting light-dependent sulfide oxidation rate measurements.

## Ocean oxygenation and ancient ocean analogs: a brief overview

Just prior to the evolution of oxygenic photosynthesis, an ocean that was fully anoxic throughout its depth existed. As oxygen production rates increased, likely driven by *Cyanobacteria* in ocean margins, oxygen would have spread from shallows and edges. This shallow layer would eventually spread and cap off deeper water layers that remained anoxic. Over time, continued photosynthetic oxygen production would produce the essentially fully oxic oceanic water column that currently exists except for **suboxic** zones (defined here are as <3 μM O2 and <0.2 μM H2S as in Trouwborst et al., 2006). This model suggests there must have existed through time, several stages of transitional oceans that were partially oxic, presumably with an oxic upper layer overlying lower anoxic layers (Figure 1).

KEY CONCEPT 2 | Suboxic Zones of very low O_2_ and sulfide concentration (<3 μ M and 0.2 μ M, respectively)

This dynamic and long term change in ocean redox conditions had profound consequences for marine chemistry and microbiology (Sleep and Bird, 2008). As ocean redox chemistry evolved, so did niches for marine microbes. For example, strictly anaerobic sulfide oxidizing phototrophic bacteria would have found progressively less suitable niche space as sulfide was pushed deeper in the water column and away from the photic zone by encroaching oxygen. The same would be true for Fe^2+^ oxidizing anoxygenic phototrophs (Crowe et al., 2008). In contrast, the increased presence of suitable oxidants (i.e., nitrate and O_2_) would have increased the available niche space for Fe^2+^ and HS^−^ oxidizing chemolithoautotrophs.

There has been considerable interest in microbial communities that reside in environments considered analogous to transitional oceans. The Black Sea (Manske et al., 2005; Marschall et al., 2010) and persistently stratified volcanic lakes (Crowe et al., 2008) serve as later stage analogs for oxygen capped **euxinic** and ferruginous systems, respectively. In contrast, Bahamian blue holes (Steadman et al., 2007; Macalady et al., 2010; Gonzalez et al., 2012) serve as possible analogs for an earlier system with a shallower oxic layer. Here, we propose the Chesapeake Bay as an additional system that likely corresponds to very late stages of ocean oxygenation.

KEY CONCEPT 3 | Euxinic Environments characterized by anoxia, restricted hydrologic circulation (i.e., stratification) and the presence of sulfide.

## The chesapeake bay system

The seasonal development of hypoxia/anoxia in the upper and middle regions of the Chesapeake Bay has been extensively documented. Low O_2_ conditions in the bottom waters are produced and maintained during the summer months by salinity stratification of the water column (Officer et al., 1984; Boicourt, 1992) and microbial decomposition of organic matter in the water column and sediments (Jonas and Tuttle, 1990; Jonas, 1992, 1997; Kemp et al., 1992) such that discrete oxic, suboxic and anoxic zones can be determined (Lewis et al., 2007). Persistent low O_2_ conditions in the bottom waters generally occur from May-September with the most pronounced oxygen depletion in late July and August (Taft et al., 1980; Malone, 1991). The severity of bottom-water suboxia/anoxia varies inter-annually with changes in the magnitudes of the spring freshet and nutrient inputs (Schubel and Pritchard, 1986; Malone et al., 1988; Harding et al., 1992; Hagy et al., 2004).

Superimposed on the general seasonal cycle of dissolved O_2_ levels are strong tidally-driven semi-diurnal oscillation, lateral seiching, and/or episodic wind-forced pycnocline disruptions (e.g., storm mixing) (Itsweire and Phillips, 1987; Breitburg, 1990; Sanford et al., 1990; Luther et al., 2004). Sanford et al. (1990), for example, collected time series measurements of dissolved oxygen (DO) and salinity at several moorings along a cross-axial transect of the mid-Bay during the summer of 1987. The strongest observed response was that due to tidal forcing. Large changes in DO and salinity at semi-diurnal and diurnal frequencies (i.e., in phase with the surface tides) were attributed to vertical movement of the pycnocline, driven by surface-forced internal tides. Longer period (sub-tidal frequency) fluctuations in DO and salinity were correlated with wind-forcing.

In addition to changes in the vertical DO profile, physical mixing processes are expected to alter the vertical distributions of other redox sensitive chemical constituents and microbial species, which depend on these chemical gradients, as well. Lewis et al. (2007) documented short-term tidal fluctuations in the depth and thickness of the suboxic layer due to tidal forcing and found episodic changes in the vertical distributions of dissolved O_2_, Mn^2+^, Fe^2+^, and H_2_S in the Chesapeake Bay water column.

Compared to our understanding of the environmental chemistry in the Chesapeake Bay, there is relatively little known about the microbiology of this system and how it changes during oxic to anoxic transitions including the dynamic short-term tidally and meterologically driven changes in chemical gradients. We are aware of only one study that has directly addressed microbial community structure during anoxia in the Chesapeake Bay (Crump et al., 2007). That study utilized denaturing gradient gel electrophoresis of 16S rDNA amplicons coupled to some chemical data to examine microbial communities at several depths in the summer of 2004. They concluded that microbial community doubling times were much more rapid than the duration of anoxia and demonstrated microbial community shifts during the onset of anoxia and increase in sulfide concentrations. While the Crump et al. study provided a general picture of microbial community shifts, little information was developed on the function of the community relative to sulfide: no relevant isolates were recovered, functional gene diversity related to sulfur oxidation was not addressed, nor were sulfide oxidation activity measurements carried out.

## Light-dependent sulfide consumption in the chesapeake bay

**Figure 2:**
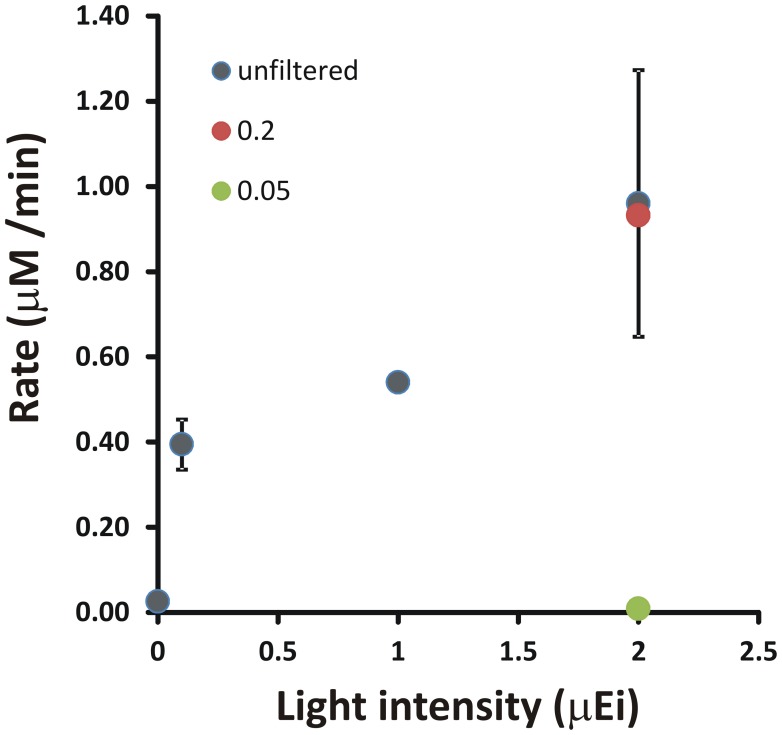
**A demonstration of light-stimulated sulfide oxidation in a shipboard incubation experiment with Chesapeake Bay waters**. Light intensity indicates the measured incident PAR for the incubation experiment. Rates were determined by linear regression of [HS^−^] vs. time for incubations under each condition. Error bars are the standard deviation of rates calculated from at least two independent incubations.

In 1988, Luther and co-workers reported water samples collected below the pycnocline in the Chesapeake Bay water column displayed sulfide consumption under anaerobic conditions (Luther et al., 1988). The key points of this initial observation were that this sulfide oxidation activity passed a 0.22 μm filter, was inhibited by treatment with formaldehyde or darkened conditions. In summer 2012, this result was essentially duplicated (Figure 2). Three specific points should be noted the 2012 data set. First, this experiment reproduced an essential component of the seminal 1988 observation, not just light-stimulated, but light-dependent anaerobic sulfide uptake. The fact that this can be observed in just two samples separated by 24 years suggests it is a normal component of the Chesapeake Bay sulfur cycle. Second, the light fluxes required to stimulate sulfide uptake activity are extremely low (<1 μ Ei m^−2^ s^−1^). If a phototrophic microbe is mediating this reaction, then it must be extremely low light adapted. For comparison, **PAR** flux on a clear summer day at noon at sea level is ~2000–2500 μ Ei m^−2^ s^−1^. Light intensities in the **hypoxic** to anoxic layers of the Chesapeake Bay are <1 to ~10 μ Ei m^−2^ s^−1^ due to depth and absorption by phytoplankton and other materials that scatter and absorb light in the photic zone. Finally, this putative microbe must also have a small cell size as the activity is essentially unchanged by 0.22 μm filtration. This small cell size and low light intensity suggest that relatively large celled cyanobacteria and algae are not likely to be sulfide oxidizers in this system. Taken together, these latter two points argue in favor of green sulfur bacteria (*Chlorobi*) as microbes responsible for this activity.

KEY CONCEPT 4 | PAR Photosynthetically active radiation.

KEY CONCEPT 5 | Hypoxic Having low dissolved O_2_ concentrations, typically <25% of saturation (<50–60 μ M), but not lacking dissolved O_2_ (anoxic).

While the <0.22 μm cell size is generally smaller than *Chlorobi* observed in laboratory cultures, *Chlorobi* are generally smaller in size than other cultured bacteria. For example *Chlorobaculum tepidum* cells are typically squat rods 0.2–0.6 μm wide by 0.4–1.0 μm long with a corresponding biovolume of 0.01–0.23 μm^3^ when grown in the Hanson laboratory (Hiras and Hanson, unpublished data). In comparison, cultured picocyanobacteria biovolumes range from 0.8 to 2.5 μm^3^ made up of cells with dimensions 1.0 μm × 1.3 μm to 1.1 μm × 2.9 μm (e.g., Haverkamp et al., 2009). Average marine bacterial cells were found to have ~0.10 μm^3^ biovolume (range −0.04–0.16 μm3, Straza et al., 2009) which corresponds to a rod shaped cell of 0.40 μm × 0.93 μm (calculated from formulas in Sun and Liu, 2003). Cells in the environment are generally smaller than cells in cultures (references in Straza et al., 2009). Since large cultured *C. tepidum* cells are similar in size to average marine bacterial cells, we expect that environmental *Chlorobi* will be smaller than average marine bacteria, and therefore a significant fraction should pass a 0.22 μm filter. In contrast, we expect picocyanobacteria to be average bacterial cell size or somewhat larger and therefore be largely removed by 0.22 μm filtration. Picoeukaryotic algae will be even larger.

As a group, the *Chlorobi* have historically been considered to be strictly anaerobic phototrophs that are confined euxinic environments that receive sunlight (Overmann and Garcia-Pichel, 2006; Imhoff and Thiel, 2010). Molecular sequence data and new isolates have now begun to challenge this view. In the marine environment, rRNA sequences related to *Chlorobium* (SAR406) have been reported from fully oxic water column samples at the base of the photic zone (Gordon and Giovannoni, 1996). SAR406-related sequences were abundant in anoxic Chesapeake Bay waters (Crump et al., 2007) and in the anoxic zone of Saanich Inlet (Zaikova et al., 2010), a seasonally stratified fjord in British Columbia. More recently, a strain of *Chlorobium* was isolated from a deep sea hydrothermal vent site (Beatty et al., 2005), where it was proposed that this strain utilizes near IR radiation emanating from hydrothermally heated minerals as a light source. This particular strain was shown to maintain full viability over 2 weeks under fully aerobic conditions in the dark and the absence of sulfide. Finally, a distinct family-level lineage of the *Chlorobi* containing a putative aerobe has been proposed on the basis of an assembled genome for “Candidatus Thermochlorobacter aerophilum,” which occurs in aerobic phototrophic mats of alkaline siliceous hot springs in Yellowstone National Park (Liu et al., 2012). These results suggest that *Chlorobi* may be able to persist between periods of anoxia or re-colonize temporally unstable anoxic regions by transport from distant anoxic environments or underlying sediments. Furthermore, extant and active populations of *Chlorobi* are found in the Black Sea chemocline (Manske et al., 2005; Marschall et al., 2010), in permanently stratified marine fjords (Schmidtova et al., 2009), and in meromictic lakes considered to be ancient ocean analogs (Crowe et al., 2008; Lauro et al., 2011). Finally, biomarker evidence indicates that *Chlorobi* may have been important components of the marine microbial community in sulfidic ancient oceans (Brocks et al., 2005). These collected observations indicate that the *Chlorobi* or other phototrophic sulfide oxidizers should play an important and previously unrecognized role in sulfide dynamics in the Chesapeake Bay.

## Why phototrophic sulfide oxidizers should matter

The redox potential of the sulfide in most environments is ~ −270 mV relative to the standard hydrogen electrode. From a microbial perspective, this means that there is substantial free energy available if the oxidation of sulfide is coupled to strong oxidants like O_2_, NO^−^_3_, Fe^3+^ or Mn^3+/4+^. However, in anoxic or suboxic marine pelagic environments, O_2_ is limited by definition, there is likely to be severe competition for NO^−^_3_ between phytoplankton and bacterioplankton as an N-source for growth that will maintain it at low levels and oxidized metals are unlikely to be found in significant amounts in the water column. The latter may not be strictly true, however, as prior measurements documented micromolar concentrations of Mn^3+^ in suboxic Cheseapeake Bay waters (Trouwborst et al., 2006). However, this condition seems to occur primarily when sulfide levels are low. Even so, the ability of chemolithotrophic bacteria to oxidize sulfide will be dependent on the availability of a suitable external electron acceptor.

Phototrophic sulfide oxidizers, through the action of photochemical reaction centers, have the ability to alter the redox potential of electrons using captured light energy, thereby altering the suite of available electron acceptors. In the case of the *Chlorobi*, reduced ferredoxin is the product of the reaction center. This is significant because the redox potential of Fd is sufficient (~ −500 mV) to reduce CO_2_. Indeed, all anaerobic *Chlorobi* are all thought to utilize the Fd-dependent reductive tricarboxylic acid cycle for CO_2_ fixation into biomass. In the case of sulfide oxidizing *Proteobacteria*, the situation is more complicated. The product of the reaction center is a reduced quinone at a redox potential of ~ −100 mV. This is insufficient to directly reduce CO_2_. However, through a process called reversed electron transport, these electrons can be donated to NADP^+^ generating reductant for the Calvin-Benson-Bassham pathway of autotrophic CO_2_ fixation, which is utilized by sulfide oxidizing phototrophic *Proteobacteria*. Chemolithotrophic *Proteobacteria* are also able to perform reverse electron transport, but without the additional energy input from light energy. Thus, phototrophic sulfide oxidizing *Proteobacteria* would be expected to out-compete chemolithotrophic sulfide oxidizers if light energy is available. What is currently unclear is if phototrophic *Proteobacteria* are able to capture the low light levels (<1 μ Ei m^−2^ s^−1^) that stimulated sulfide oxidation in Chesapeake Bay samples (Figure 2). At this point, what we can say is that culturing efforts carried out in 2011–2013 only yielded *Chlorobi* in enrichments for phototrophic sulfide oxidizers from Chesapeake Bay water column samples (Findlay et al., unpublished data).

## Techniques for measuring sulfide oxidation rates

To fully understand the potential role of phototrophic sulfide oxidizers in the Chesapeake Bay and elsewhere, the kinetics of light-dependent sulfide oxidation must be determined (Figure 2), both in environmental samples and laboratory cultures. This necessarily requires the accurate measurement of sulfide concentrations over time courses in samples. The bisulfide anion (HS^−^) is the predominant form of dissolved sulfide at neutral to mildly alkaline conditions (H_2_S <–> HS^−^ + H^+^, pKa ~6.5) in most marine environments. A broadly applicable method for kinetic analysis of sulfide oxidation in both aerobic and anaerobic microbial systems should provide real-time or near real-time data and have high sensitivity. A further consideration is whether a technique is able to detect more than one sulfur species including the sulfide oxidation products polysulfides and thiosulfate. The ability to measure these oxidized sulfur species enables direct detection of precursor-product relationships and allows one to distinguish between oxidation and cellular uptake.

The bisulfide anion in solution can be measured directly by its UV absorbance ~231 nm and it can be measured in natural waters even in the presence of dissolved organic materials by spectral deconvolution (Guenther et al., 2001; Luther et al., 2011). However, the presence of microbial cells in kinetic oxidation assays limits the effectiveness of this method due to a high degree of UV absorption and light scattering. The classic method for determining dissolved bisulfide is the Cline or methylene blue assay (Cline, 1969) that relies on the conversion of a colorless precursor to methylene blue in the presence of sulfide. This assay is reliable and has been used extensively in natural systems (e.g., Zopfi et al., 2001) and with phototrophic sulfide oxidizers (e.g., Arieli et al., 1991). However, the Cline assay can be cumbersome for kinetic experiments because rapid sacrificial subsampling of an assay mixture is required. This subsampling in turn changes assay volume which can cause changes in culture illumination for phototrophic microbes. It is also sensitive to interference by polysulfide, the first oxidation product of sulfide, leading to an underestimation of sulfide concentrations (Luther et al., 1985). More recently bimane derivatization has been applied to measure sulfide and other thiols in natural samples (thiol = R-HS-, Rethmeier et al., 1997). While bimane is more sensitive than the Cline assay and also detects sulfite, polysulfides, and thiosulfate, it again requires sacrificial sampling and sample processing. Therefore, it does not provide a real-time read-out of activity. Bimane methods also require significant instrumentation (a gradient capable HPLC with fluorescence detector) to be implemented. For real-time data, respirometric methods (i.e., quantifying sulfide-dependent O_2_ uptake, Gonzalez-Sanchez et al., 2009) can be utilized for aerobic sulfide oxidizers, but not for anaerobes.

Based on the above considerations, electrochemical methods of sulfide detection fulfill most of these requirements and have been applied both *in situ* for environmental/water column analysis and in the laboratory to measure sulfide oxidation kinetics in samples and cultures. These methods are particularly appropriate when many short-duration (i.e., minutes to hours) assays need to be performed with high data collection (i.e., several times per minute or more) rates are required to capture initial rates of sulfide oxidation. General reviews on the use of electrodes and electrochemical techniques in microbial ecology and as *in situ* sensors have been published elsewhere (Revsbech, 2005; Moore et al., 2009). To build upon the observations described above and in our previous Frontiers manuscript (Luther et al., 2011) required that a common set of protocols be developed so that comparable data sets for phototrophic sulfide oxidation kinetic measurements could be collected in several physically distinct laboratory settings by different personnel. Here, we describe the basic apparatus and document the variability in these measurements so that other groups wishing to utilize these methods may do so more readily. In doing so, our goal is to provide a solid technical foundation for others that wish to make similar measurements and allow them to benefit from our experiences in setting up these types of experimental rigs.

## Electrochemical measurement of phototrophic sulfide uptake

The apparatus for measuring phototrophic sulfide oxidation by electrochemical methods is relatively straightforward: an electrochemical analyzer system equipped with gold- amalgam (Au/Hg) electrodes, a clear temperature controlled electrochemical cell that can be made anoxic, a light meter equipped with a quantum PAR sensor, a controlled illumination source and a shroud to exclude extraneous light. The details of electrode fabrication have been presented in detail elsewhere (Brendel and Luther, 1995; Luther et al., 2008) and will not be described here.

**Figure 3:**
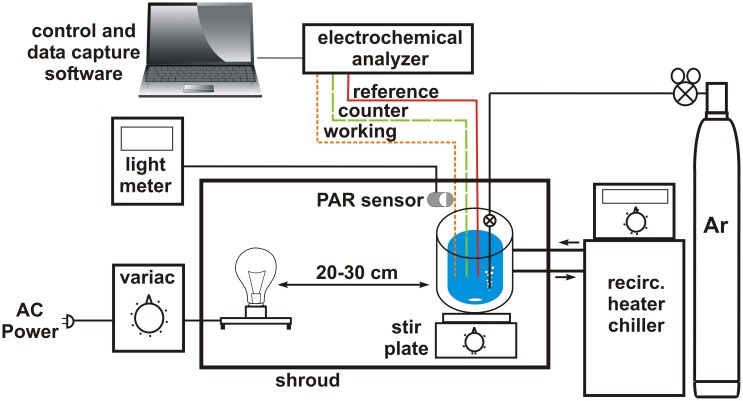
**Schematic of experimental apparatus for reproducibly measuring phototrophic sulfide uptake**. Details for particular components are provided in the text. “Reference,” “counter,” and “working” refer to the electrodes of the electrochemical analyzer system.

The schematic for arranging these components is shown in as general a fashion as possible (Figure 3). The specific brand/manufacturer of the components should not, in principle, affect the outcome of the experiment. The electrochemical cell, light source, and light sensor are contained inside the shroud with cables, gas and water (for temperature control) lines traversing the shroud. The electrochemical analyzer, control and data capture computer, light meter, temperature control (recirculating heater/chiller) and gas source are all located outside of the shroud. In principle, the shroud can be any light blocking fabric or non-reflective material (i.e., velvet, cardboard, etc.). For ease of manipulation, a frame constructed of stock laboratory stands and crossbars can be draped with multiple layers of black plastic sheeting (available at local hardware or paint stores) secured to the frame by binder clips. The flexibility of the plastic allows it to be overlapped and taped where cables and lines penetrate the shroud to minimize light leakage. A variac (voltage attentuator) connected to a single bulb fixture provides variable light intensity. Full spectrum incandescent bulbs (i.e., G.E. Reveal) ranging from 10 to 60 W output provide a broad range for low and high light intensity experiments. Using this apparatus, we can routinely obtain a steady light field of 0.1 μ Ei PAR (~0.005% of summer noontime light intensity). For most purposes this will be low enough light intensity. However, for extremely low light intensity, i.e., for replicating light levels at the upper reaches of the Black Sea euxinic zone (0.0008–0.0022 μ Ei, Manske et al., 2005), utilizing this apparatus in a darkened room and with higher sensitivity light meters/sensors may be necessary.

The electrochemical cell is attached to an Ar supply so that O_2_ can be excluded during measurements. Ar is preferable to N_2_ due to its higher density giving it the ability to “cap” the solution in the electrochemical cell. For work with pure cultures, collecting cells for assay under anoxic conditions is also important as is the buffer used for maintaining pH during the assay. After experimenting with various buffering systems, we have found that anoxic 0.1 M HEPES at pH 7.4 provides effective pH control without introducing any background signals during cyclic voltammetry. Cells are typically washed twice by centrifugation (with all transfers to centrifuge bottles carried out in an anaerobic chamber or glove bag) and resuspended in HEPES buffer to remove any medium components and external substrate.

For any given measurement, the empty electrochemical cell is first flushed with Ar, followed by the addition of anoxic HEPES buffer or environmental sample and a short period to allow for equilibration to temperature (i.e., for the moderate thermophile *C. tepidum*). At this point scans are performed to verify that the cell is anoxic (i.e., no observable O_2_ and H_2_O_2_ peaks in the voltammogram). If necessary, the solution is purged with Ar until no O_2_ is detectable. This is followed by the addition of washed cell suspension. The assay is then initiated by the addition of sulfide to a defined concentration. Typical working volume for an assay is ~20 ml in the electrochemical cells employed in our laboratories. The suspension in the electrochemical cell is continually stirred except when cyclic voltammograms are being collected (~every 15–60 s). A critical control is to measure the sulfide loss rate from cell-free buffer to determine if there is a significant abiotic loss component in the apparatus. One potential cause is poor sealing of the electrochemical cell that allows volatile sulfide to escape. This loss rate should be <1% of the rate observed in the presence of cells. To compare between samples, the initial rate of sulfide loss is determined by linear regression of the sulfide concentration values. In the case of cultures, this rate is then normalized for the amount of biomass to provide a biochemical specific activity, i.e., the rate of sulfide oxidation catalyzed by each unit of biomass in the assay. To collect kinetic data and compare cells under different environmental parameters (i.e., varying light intensity or HS^−^ concentration), it is important to maintain biomass at as constant a level as possible between assays near the top of the linear range of activity vs. biomass (i.e., see Figure 6 in Luther et al., 2011) so that small fluctuations in sulfide uptake rate can be sensitively and reproducibly detected.

**Figure 4:**
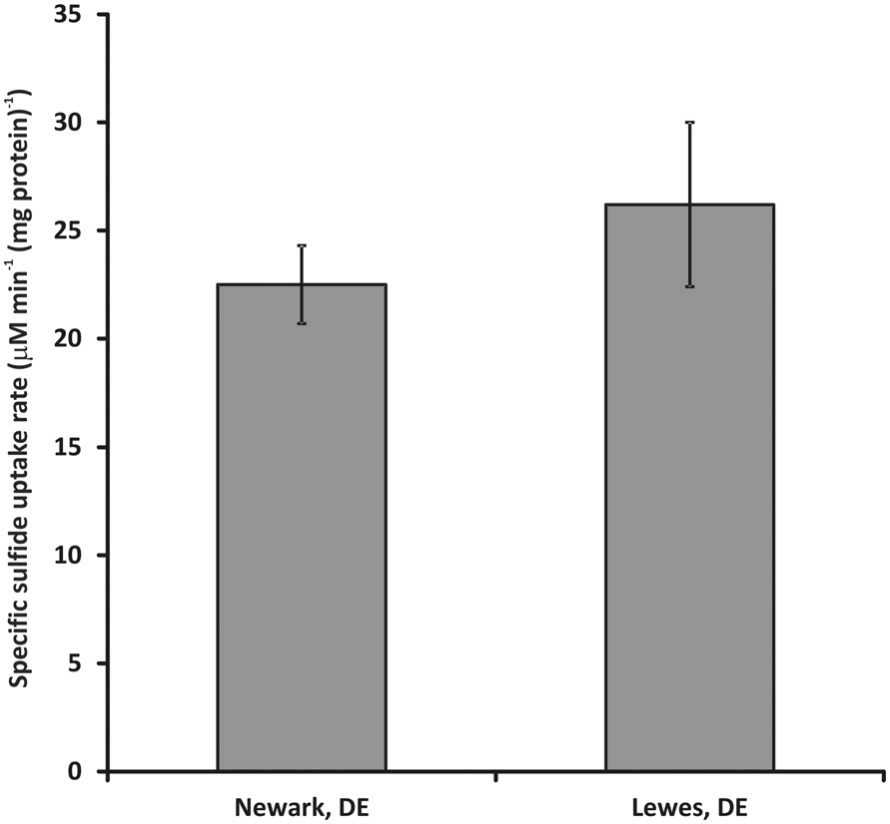
**Inter-laboratory comparison of sulfide uptake rates for washed cell suspensions of *C. tepidum***. Sulfide uptake rates were assayed at 50–60 μ M initial HS- concentration at 5 μ Ei m^−2^ s^−1^ PAR flux with a biomass concentration of 5 (μ g protein) ml^−1^. Error bars represent the standard error of the data set (*n* = 10 for Lewes, DE and 27 for Newark, DE).

When the procedures outlined above are taken, directly comparable results that display no significant statistical difference can be produced by different personnel in different laboratories (Figure 4). Note that this does not mean the data produced are identical, as can be seen from the magnitude of the error bars in the inter-laboratory comparison. This noise in the data set may derive from subtle differences in strain growth conditions between the two laboratories or experimental apparatus. For example, the Lewes laboratory typically employs an automated cell holder while the Newark laboratory uses manual control of cell stirring. However, the magnitude of the variance is similar between the two laboratories suggesting that the equipment differences do not impart significantly greater variance in one laboratory vs. the other.

A final caveat is that the apparatus here and its use as described measures the disappearance of sulfide from solution, which is distinct from sulfide oxidation and could also be due to passive loss due to volatilization (e.g., DeLeon et al., 2012). Assuming that control measurements document that passive loss is not occurring, the absence of additional evidence for the production of intermediate oxidation state sulfur species (i.e., polysulfide, S(0), thiosulfate, etc.), requires that the biologically driven sulfide loss be interpreted as sulfide uptake. As noted above, the ability to readily detect polysulfide and thiosulfate using the electrode systems outlined here allows for the possibility of discriminating uptake from oxidation and establishing precursor-product relationships. Other electrodes, for example Ag/Ag_2_S electrodes for total S^2−^ activity (e.g., described in Revsbech et al., 1983 and Revsbech, 1989) that can provide good sulfide sensitivity are limited in this aspect and can be subject to other interferences as discussed elsewhere (e.g., Kühl et al., 1998). The alternative amperometric electrode system described by Kühl et al. (1998), similarly is unable to provide information on more oxidized sulfur species and therefore unable to distinguish directly between oxidation and passive loss or uptake.

## Concluding remarks

This focused review has tried to convey the current understanding of sulfidic marine systems as ancient ocean analogs. In these environments, phototrophic sulfide oxidizers should have thrived, including members of the *Chlorobi*. Furthermore, current and past data from the Chesapeake Bay indicate that *Chlorobi* may have more significant roles to play in modern, transiently anoxic marine ecosystems than is currently appreciated, though there are still many experiments that need to be performed to rigorously test this hypothesis. To facilitate these experiments, we have outlined techniques to measure phototrophic sulfide consumption rates at low light intensities with both cultures and environmental samples.

### Conflict of interest statement

The authors declare that the research was conducted in the absence of any commercial or financial relationships that could be construed as a potential conflict of interest.
